# Orthogonal-view microscope for the biomechanics investigations of aquatic organisms

**DOI:** 10.1016/j.ohx.2024.e00533

**Published:** 2024-04-22

**Authors:** Brian T. Le, Katherine M. Auer, David A. Lopez, Justin P. Shum, Brian Suarsana, Ga-Young Kelly Suh, Per Niklas Hedde, Siavash Ahrar

**Affiliations:** a Department of Biomedical Engineering, California State University Long Beach, 1250 Bellflower Blvd. Long Beach, CA 90840, USA; b Beckman Laser Institute and Medical Clinic, University of California Irvine, Irvine, CA 92612, USA

**Keywords:** DIY microscope, Imaging, Orthogonal view, Biomechanics of organisms

## Abstract

Microscopes are essential for the biomechanical and hydrodynamical investigation of small aquatic organisms. We report a prototype of a do-it-yourself microscope that enables the visualization of organisms from two orthogonal imaging planes — top and side views. Compared to conventional imaging systems, this approach provides a comprehensive visualization strategy of organisms, which could have complex shapes and morphologies. The microscope was constructed by combining custom 3D-printed parts and off-the-shelf components. The system is designed for modularity and reconfigurability. Open-source design files and build instructions are provided in this report. Additionally, proof-of-use experiments (particularly with *Hydra*) and other organisms that combine the imaging with an analysis pipeline were demonstrated to highlight the system’s utility. Beyond the applications demonstrated, the system can be used or modified for various imaging applications.

## Graphical abstract

**Figure:**
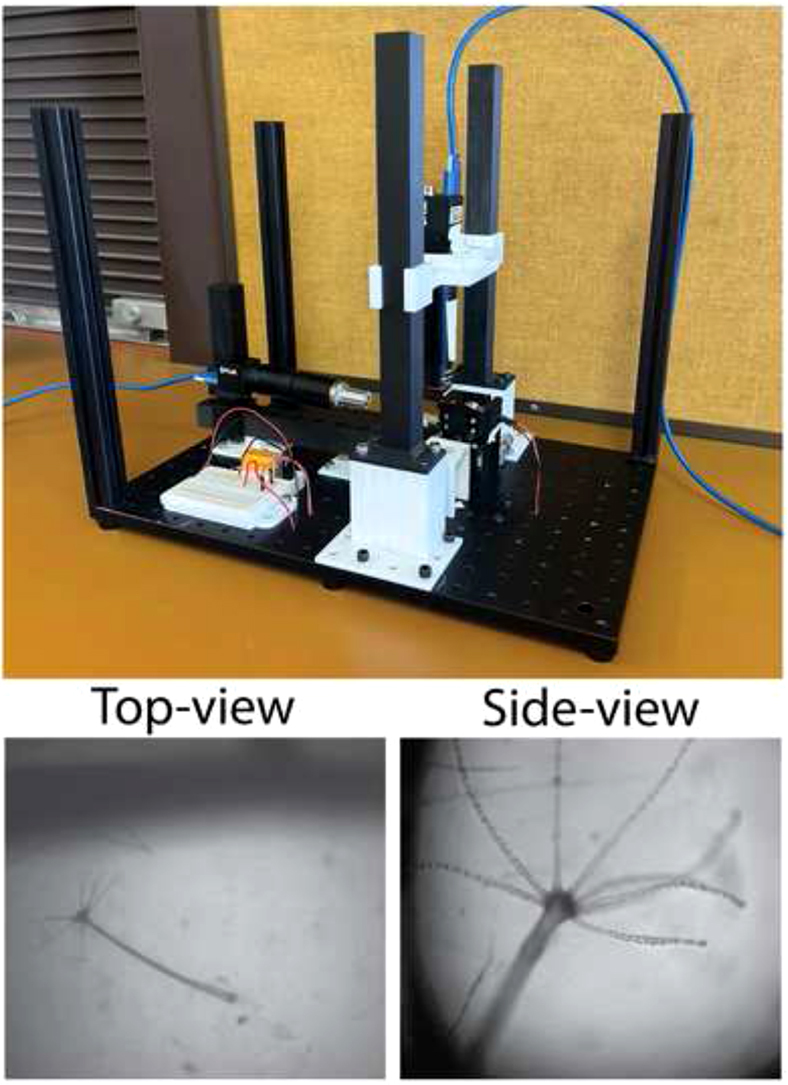

## Specifications table

**Table:** 

Hardware name	*GLUBscope: the Orthogonal-view Microscope*
Subject area	• *Engineering and material science*
Hardware type	• *Imaging tools*
Closest commercial analog	• *No commercial analog is available*
Open source license	• *CC-BY-SA 4.0*
Cost of hardware	• *$ 3000–4000*
Source file repository	https://doi.org/10.17605/OSF.IO/UAJXB

## 1 Hardware in context

Investigating biomechanics [1], [2], [3], hydrodynamics [4], and mechanobiology [5] of organisms is of great interest. Beyond their fundamental biological importance, these investigations could provide approaches to mitigate or address the emerging threats to biodiversity due to climate change [6]. Therefore, broader access to imaging tools, specifically microscopes, could be critical for laboratory and field investigations. Unfortunately, most commercially available microscopes are expensive, difficult to deploy for field studies, and challenging to customize [7]. Thankfully, microscopes have benefited from the emergence of the do-it-yourself (DIY) and open hardware movement. The DIY movement has reduced costs, provided access to local fabrication, allowed for customization, and increased access to imaging instruments [8]. This report describes an open-sourced DIY orthogonal-view microscope for biomechanics and hydrodynamics investigation of aquatic organisms. Over the past two decades, significant advancements have been made in developing low-cost microscopes and other DIY hardware. Salido et al. comprehensively summarize these advances in their review [9]. Del Rosario et al. provide key considerations for constructing microscopes using 3D printing [10]. Many creative examples of DIY microscopes have been reported [11]. For example, the OpenFlexure project integrates optics with 3D-printed precision mechanical positioning, enabling automated microscopy accessible to a wide range of users [12]. In another example, the UC2 (You. See. Too.) initiative has developed a modular and open-source toolbox that facilitates the construction of DIY microscopes [13]. Moreover, researchers have successfully produced 3D-printed components enabling imaging and control of model organisms (e.g., temperature) [14], as well as microscopes tailored for controlling optofluidic applications [7], DIY incubator-contained microscope (i.e., Matchboxscope [15]), and a low-cost automated scanning microscope using the mechanical components of a 3D printer (for example, EnderScope [16]). In the study of marine organisms, PlanktoScope [17], [18] and the scale-free tracking microscope [19], [20] are of particular interest. Additionally, the use of mobile phones in conjunction with DIY hardware has been successful [21], [22], [23], [24]. However, an imaging system that enables the visualization of a sample from multiple sides has remained absent.

In this investigation, we developed a proof-of-concept orthogonal-view microscope for biomechanics investigations of aquatic organisms. The microscope, nicknamed GLUBscope, was built by combining custom 3D-printed parts and broadly available off-the-shelf components. The key feature of the microscope is providing two orthogonal planes of imaging - a top view and a side view (90°) from each other. Orthogonal imaging is a commonly used approach in X-ray imaging as part of diagnostics to visualize features from two independent perspectives. In our efforts, imaging provided an avenue to see an organism even when it moves out of a plane in one view. Due to their complex 3D shapes, viewing organisms from multiple angles could be critical in addressing questions related to biomechanics and hydrodynamics (e.g., surface attachment, multi-dimensional elongation, or stretching in multiple planes). We avoided the immobilization strategies and used chambers (3.5 mL volume) with larger volumes than the standard microfluidics often used for biomechanics investigations. The system was built for modularity and reconfigurability, such that various components could be readily exchanged. This report represents a proof-of-concept for this orthogonal and simultaneous imaging. The current design has the potential to improve further by implementing additional features such as automated mechanical sample positioning, automated tracking, or 3D reconstruction of organisms. In this report and accompanying repository, we have shared design files, bill of materials, and build and operation instructions. Additionally, proof-of-use experiments with organisms (particularly *Hydra*) are provided to demonstrate the use of GLUBscope for biomechanics investigations.

## 2 Hardware description

The GLUBscope provides two orthogonal views of the samples, which is unique as compared to conventional microscopes. The GLUBscope was built using off-the-shelf components and DIY 3D-printed parts. The system was built on an optical breadboard measuring 12” × 18” × 1/2”. Almost all commercial mechanical components (e.g., optical breadboard) can be replaced with alternatives depending on their use.

**Fig. 1:**
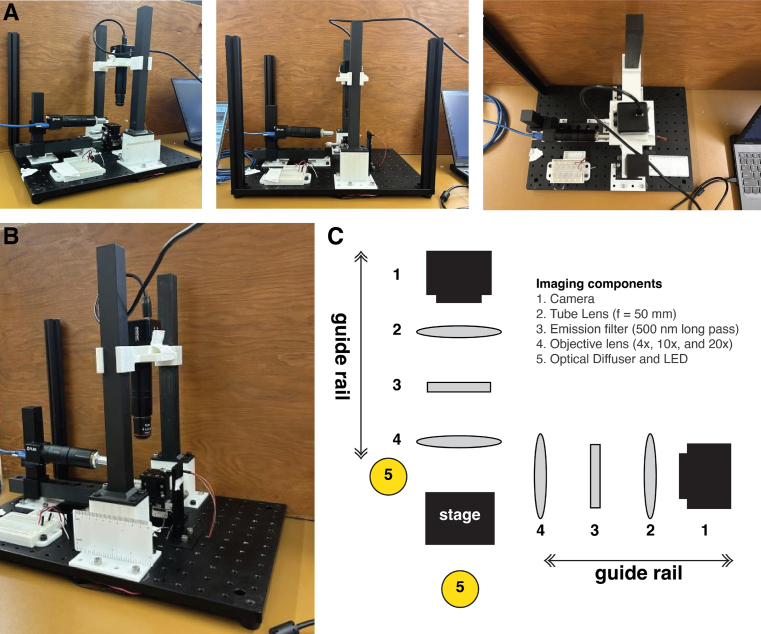
**GLUBscope - system overview**. (A and B) Photographs of the assembled GLUBscope. In this configuration, two different types of cameras were used. (C) System block diagram of the primary optical components.

**Design rationale**: Two imaging arms (containing a camera, a tube lens, an emission filter, and an objective lens) were used for each imaging plane. These arms were positioned on 3D-printed manual guide rails. Given the proof-of-concept nature of this effort, manual control for positioning was used. The objective lens for each arm could be independently adjusted. The guide rails were used to rough-position the samples. An XYZ micromanipulator stage was used to fine-position the samples. Fig. 1 presents images and a simplified system block diagram emphasizing the optical components. The two guide rails to move optical components to focus the image were built using Snapmaker 2.0 A350T Modular 3-in-1 3D Printer and Markforged Mark Two 3D Printer. The guide rails could bring the objectives sufficiently close to the sample to enable fine focusing with the stage. Two cameras were used, one for capturing the top view (or vertical view) and another for the side view (or horizontal view). We used two (20 MP)/high-sensitivity cameras (FLIR) or two Arducam cameras, offering a choice between high-resolution/high-sensitivity and more cost-effective options. Camera control was achieved using Spinnaker SDK, Micromanager (both for FLIR hardware), or AMCap software (for Arducam cameras). The study used various objectives ranging from 4X to 10X to enable different magnification requirements. Brightfield imaging of the samples was achieved by employing two bright, white LEDs positioned below and on the side of the sample. A stand was developed for side illumination. An optional diffuser holder was developed for top view illumination. With portability in mind, LEDs were powered by a 9 V battery and regulated using two resistors (variable or fixed at 350 Ω) such that the system could be operated in the field without access to an electrical grid. The components were secured onto the optical breadboard via 1/4”-20 screws. The bill of materials, the designs for the 3D-printed parts, and other resources are provided through an Open Science Framework (OSF) page.

Potential uses of the GLUBscope include:

• *Orthogonal view imaging (top and side).*
• *Biomechanics and hydrodynamics investigations of aquatic organisms.*
• *Field applications.*
• *Pedagogy or classroom applications.*

## 3 Design files summary

Designs for the 3D-printed components in SLDPRT format are available from the supplemental materials and the project’s Open Science Framework repository, https://doi.org/10.17605/OSF.IO/UAJXB. The following is a summary of the hardware files.

**Table:** 

Design filename	File type	License	Location of the file
breadboard_and_battery_holder	3D Model (.sldprt)	CC-BY-SA 4.0	OSF page
horizontal_camera_holder	3D Model (.sldprt)	CC-BY-SA 4.0	OSF page
horizontal_camera_path	3D Model (.sldprt)	CC-BY-SA 4.0	OSF page
horizontal_camera_post	3D Model (.sldprt)	CC-BY-SA 4.0	OSF page
horizontal_camera_stands	3D Model (.sldprt)	CC-BY-SA 4.0	OSF page
horizontal_light	3D Model (.sldprt)	CC-BY-SA 4.0	OSF page
vertical_camera_adapter	3D Model (.sldprt)	CC-BY-SA 4.0	OSF page
vertical_camera_holder	3D Model (.sldprt)	CC-BY-SA 4.0	OSF page
vertical_camera_posts	3D Model (.sldprt)	CC-BY-SA 4.0	OSF page
vertical_camera_stands	3D Model (.sldprt)	CC-BY-SA 4.0	OSF page
vertical_camera_flir	3D Model (.sldprt)	CC-BY-SA 4.0	OSF page
diffuser holder v2	3D Model (.sldprt)	CC-BY-SA 4.0	OSF page

The supplementary material provides an animated rendering of the components’ assembly.

## 4 Bill of materials

A bill of materials is available from the OSF page (please see under Wiki) and provided as support files with additional comments. Potential vendors are suggested. We note that availability and costs are subject to suppliers. Alternative options are recommended when possible.

## 5 Build instructions

**Fig. 2:**
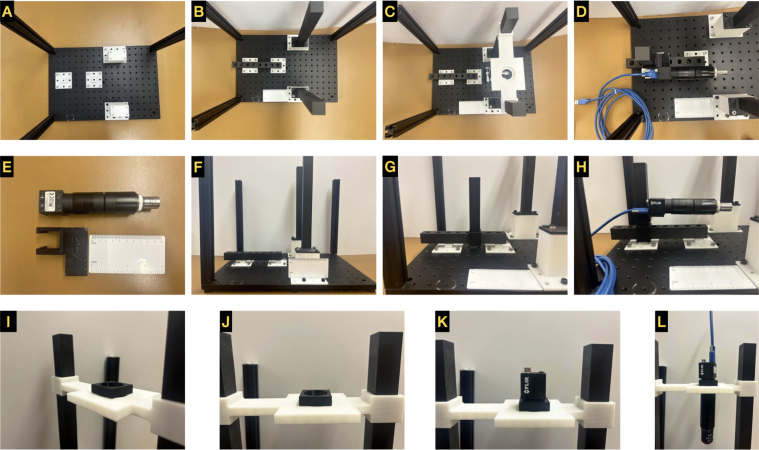
**Build instructions part 1: hardware and optics.** (A: C) Positioning of components (posts and guide rails) for imaging. (D: H) Side-view imaging components. (I: L) Top-view imaging components.

**Fig. 3:**
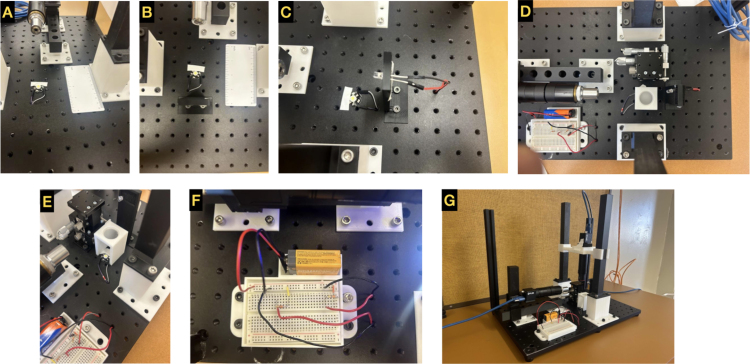
**Build instructions part 2: LEDs, electronics, and stage**. (A: E) LEDs for vertical and side illuminations. The use of the diffuser and holder are highlighted in images D and E. In this example, bright field LEDs are demonstrated. (F) Electronic components to power the LEDs. (G) Fully assembled GLUBscope.

Step-by-step instructions for the system build, and images are provided (Fig. 2, Fig. 3). Please note that for the connections listed below relying on 1/4” - 20 screws, it is possible to reverse the orientations while constructing the GLUBscope with no negative impact on build stability. Four screws are the minimum requirement for a secure connection between the base of a part and the optical breadboard. There are additional ports available for a more secure attachment. A degree-marked level (circular bubble level bullseye) can be used to ensure that the parts are level. During the system build, we urge the following safety guidelines to be considered.

■ SAFETY 1: Please use proper personal protective equipment (PPE). Bright LEDs and other light sources could pose a safety risk.
■ SAFETY 2: Recall that the extruder and print bed of a 3D Printer can become hot. After the print is completed, remove the part from the bed by applying a gentle shear force. If a chisel is required to remove the part, please use appropriate PPE, such as cut-proof gloves and safety glasses.
■ SAFETY 3: Please use proper ventilation during 3D printing to avoid inhaling small particles or toxic fumes.
■ SAFETY 4: Please use appropriate ventilation and PPE when soldering.

The following are the instructions for building the GLUBscope.

**Horizontal Path Assembly**
  (a) Place the M6 breadboard on a flat surface. The position (port) on the top left corner is referenced as coordinate (1,1).
  (b) Optional Step: Apply double-sided (set) screws in the 4 corners (1,1),(1,12),(18,1),(18,12). Attach optical construction rails to create an enclosure for the system to block ambient light.
  (c) Roughly align the **vertical_camera_stands** in desired coordinates (9,1),(9,10), spaced 166 mm apart or with 6 ports spaced between them.
  (d) Secure the stands via four screws.
  (e) Align **horizontal_camera_stands** centered at coordinates (3,7),(8,7), spaced 58.44 mm outside to inside.
  (f) Fasten screws into 4 corners. See Fig. 2 **A**.
  (g) Place the **horizontal_camera_path** on top of **horizontal_camera_stands** and secure in place with screws. See Fig. 2 **B**.
  (h) Slide **horizontal_camera_posts** into the **horizontal_camera_path**. See Fig. 2 **D and E**.
  (i) Attach the FLIR camera with optical components (See Fig. 2 **E**) to the **horizontal_camera_holder**.
  (j) Slide the **horizontal_camera_holder** with a camera attached to the **horizontal_camera_post**.
  (k) Connect the FLIR camera to USB 3.1 Gen 1 Micro-B to USB-A Locking Cable and then the computer. See Fig. 2 **H**.
**Vertical Path Assembly**
  (a) Align **vertical_camera_posts** with **vertical_camera_stands** and secure in place with screws.
  (b) Slide **vertical_camera_holder** on **vertical_camera_posts**, with the short side closest to the user.
  (c) Place the **vertical_camera_adapter** on the top of the **vertical_camera_holder** and align the ports. See Fig. 2 **I and J**.
  (d) Align the FLIR camera in the **vertical_camera_adapter_flir**. See Fig. 2 **K**.
  (e) Attach the optical path with the desired lens, filter, and objective on the bottom port of **vertical_camera_holder** Connect the FLIR camera to a computer via USB 3.1 Gen 1 Micro-B to USB-A Locking Cable. See Fig. 2 **L**.
  (f) Alternatively, the Arducam camera can be used. A USB 2.0 cable was used to connect the camera to the computer.
**Lighting System Assembly**
  (a) Solder jumper wires to the 3 W LEDs (flat form factor) for vertical illumination. Please note that one or two LEDs may be needed. LEDs with a standard form factor can be used for the side illumination. These LEDs can be directly attached via jumper wires.
  (b) Attach the 3 W LED on the optical breadboard. Roughly align the LED with the objective for vertical imaging between (12,6) and (12,7). See Fig. 3 **A**.
  (c) Run wires underneath the optical breadboard in port (13,6). The electrical breadboard connects these wires to the circuit and 9 V battery.
  (d) Use foam materials inside the rectangular opening of the **horizontal_light** to secure the side illumination LED. Then, attach **horizontal_light** to the optical breadboard via (14,5),(14,6). Fig. 3 **B**.
  (e) Connect the LEDs to jumper cables. Roughly align the LED with the horizontal objective.
  (f) Move the wires underneath the optical breadboard via (15,6) to be later connected to the electrical circuit.
  (g) Attach the **breadboard_and_battery_holder** to the optical breadboard via (3,2),(3,3) and (7,2),(7,3) positions.
  (h) Attach the Micromanipulator stage into ports (11,8) and (13,8).
  (i) Place the electrical breadboard and a 9 V battery in their respective holders.
  (j) Using two 350 Ω resistors (or two potentiometers) and necessary wires, create two series circuits to power the LEDs via the 9 V battery. See Fig. 3 **F**. It is important to select resistors (or two potentiometers) with appropriate power ratings.
  (k) Connect the wires from both LEDs to the electrical circuit above the optical breadboard through ports (8, 3) and (8, 2). See Fig. 3 **F**.
  (l) Place the **diffuser holder v2** above the 3 W LED for vertical illumination. See Fig. 3 **D and E** for the position.
  (m) Place an optical diffuser inside the **diffuser holder v2** for better illumination.
  (n) Optional light features (e.g., 360 Flexible Gooseneck Clamp Lamp) can be used for alternative illuminations.

**Fig. 4:**
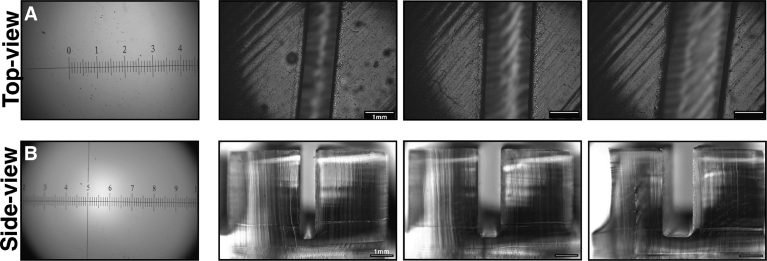
**System verification via standard slides and polymer channels**. (A) Reference slide 1, viewed from the top view, and a set of open channels (as a reference) with increasing channel width. (B) Reference slide 1 viewed from the side with increasing channel widths. Channels were created by casting PDMS from 3D-printed molds.

## 6 Operation instructions

### 6.1 Camera configurations

The GLUBscope cameras can be operated with one or two computers. When using the two high-resolution (20 MP)/high sensitivity cameras (FLIR), a single computer, and the control software from the manufacturer (Spinnaker SDK) were used. When using two different cameras, two independent computers were used. Using two computers also simplified memory management. High-resolution cameras were connected via a USB 3.1 Gen 1 Micro-B to USB-A locking cables to computers. High-sensitivity cameras can be controlled with the Micromanager software. We have provided guides for the configuration and setting selections while using Micromanager in the OSF repository. We also verified that for the more economical option, Arducam cameras (webcams) could be used. These cameras were connected with a USB 2.0 cable to the computers. The Arducam cameras were controlled via the AMCap software. These options demonstrate that camera selection can be configured based on a specific application.

### 6.2 Sample positioning

All components should be level for the best alignment. Next, turn on the LEDs to confirm that the cameras can collect live streams. The guide rails are used for rough focus, and the XYZ micromanipulator stage is used to fine-focus the sample. Typically, the edge of a slide or a cuvette can be used as a target for focusing an image. Please note that the position of the light sources can be adjusted to better illuminate the entire field of view. In our experience, if the LEDs were too close to the sample, only a portion of the field was illuminated. The diffusers (for the top view) improved the illumination. Please note that simultaneously viewing the same field of view (top and side) may require adjustments. A Flexible Gooseneck Clamp Lamp can be used for easily adjusting the illumination of a sample. Using a lower magnification for the top view was helpful to this aim. Our investigations used a 4X objective for the top view and a 10X for the side view. The working distance of the objectives was not an issue. This parameter should be considered for higher magnification objectives. Objectives can be changed based on a project’s needs. Additionally, LEDs can be adjusted – the distance between the light source and the sample and brightness – to better illuminate the sample.

**Fig. 5:**
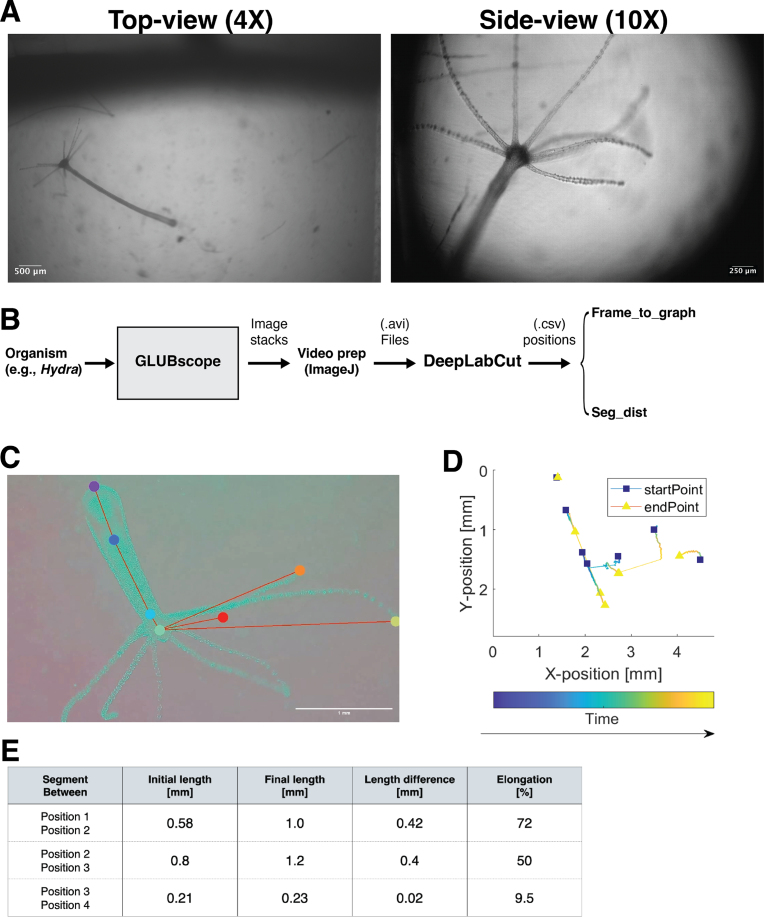
**GLUBscope - *Hydra* case study**. (A) *Hydra* were visualized from both top and side views. (B) Approach for tracking anatomical features (C: D) Tracking features via DeepLabCut. The plot demonstrates the anatomical features’ starting and final position. (E) Biomechanical analysis to estimate segment lengths and elongations.

### 6.3 3D printing

We produced prototype components using a Snapmaker 2.0 A350T Modular 3-in-1 3D Printer with Polylactic Acid (PLA) filaments. The finalized version of guide rails were printed with a Markforged Mark Two 3D printer with nylon composites to improve mechanical durability.

## 7 Validation and case studies

**Calibrations**: We utilized a calibration slide as a 2D standard object and a rectangular channel as a 3D standard object to test calibration capacity. Standard slides (i.e., a 0.1 mm calibration slide) were used to obtain top and side images. Images generated from the references are presented in (Figs. 4**A and B**). Please note that the top and side view of 0.1 mm is illuminated via an LED with a diffuser. Here, the channel profiles could be easily visualized from each direction. The channels were created by casting silicone (PDMS) from 3D-printed molds. The channel side view were illuminated using an LED lamp. GLUBscope was also used as a fluorescence microscope. To this aim, the white LEDs were switched with bright green LEDs. 5μm green fluorescent beads were used as the sample. Beads were suspended in water and were added to the cuvettes (3.5 mL volume, 10 mm path length, 4 Clear Windows). Glubscope successfully captured the bead clusters within the region of interest according to the intensity profile along the sample line. Preliminary results are presented as supplementary material (**SupFigure 1**). The fluorescence feature requires further optimizations to improve the excitation light source.

**Organism biomechanics**: Proof-of-use experiments were conducted to demonstrate using GLUBscope in biomechanics investigations. In the first demonstration, freshwater polyps (*Hydra vulgaris*) were imaged using the top-view (4X) and side-view (10x) (see Fig. 5**A**). A simple analysis pipeline was developed to enable biomechanical investigations (see Fig. 5**B**). Using the video recordings from the microscope and DeepLabCut application [25], anatomical landmarks from the organism were tracked. These included features such as the foot, two points across the body column, tentacles, and the head (see Fig. 5**B**). In the presented position plot, blue squares represent the starting position, and yellow triangles mark the final position. Changes in position are highlighted by lines connecting these two shapes with color coding encoding time. Moreover, two simple applications, *Frame to segment* and *Segment to distance*, were developed for additional biomechanical analysis. Using the position data generated by DeepLabCut, initial and final length segments and percentage elongation of the segments were calculated. The source code for both applications is available from the repository.

Next, GLUBscope was used to visualize sand dollar larvae (*Dendraster Excentricus*) and sea anemones (see **SupFigure 2**). For larvae, GLUBscope enabled the imaging of the complex structure of the larvae from multiple angles. While preliminary, this ability could provide a unique approach to visualizing the complex hydrodynamics of these organisms. Side visualization was critical for the final demonstration, imaging sea anemones since the organisms were attached to an opaque material that blocked light transmission from the top view. Collectively, the three proof-of-use experiments demonstrate various applications of GLUBscope to study aquatic organisms.

## 8 Conclusion

We demonstrated GLUBscope, a proof-of-concept orthogonal view microscope. The system was modular and made via DIY 3D-printed parts and off-the-shelf components. GLUBscope was designed to enable imaging of samples from two orthogonal planes. This feature is useful for biomechanical investigation of aquatic organisms. We primarily used brightfield illumination throughout the study (via LEDs or LED lamps). Preliminary fluorescent imaging was also demonstrated. Brightfield illumination was used for most of the study, and preliminary fluorescent imaging was also demonstrated. Better sources of illumination (brighter LEDs, or laser) similar to our prior efforts are needed [26] to advance this feature. To record from two planes of imaging, the implementation required two cameras. We demonstrated the use of high-resolution/high-sensitivity and more economical options. This demonstration is valuable since the cameras are the most expensive component. As part of the report, proof-of-use experiments and analysis pipelines (specifically biomechanical studies of *Hydra*) were demonstrated. Organisms in the current system were housed inside a cuvette (3.5 mL, 10 mm path) with four polished sides. In future experiments, microfluidics or other customized flow chambers, as described in our previous efforts [27], could be employed. It is important to note that investigating certain aspects of the hydrodynamics of freely moving aquatic organisms (e.g., sand-dollar larvae) may require modifications, larger volumes, and complex flow microenvironments. Given the proof-of-concept nature of this report, there are many areas for future improvements. These include automated sample positioning and fusing of images (which was not the primary goal of these efforts). Additionally, the development or use of existing software that enables 3D tracking and automated image synchronization (currently achieved manually) are needed to extend the use of the system.

Among the existing DIY microscopes, the scale-free tracking system [19], [20] is an elegant approach for providing a free path for the movement of organisms in one direction. However, conventional chambers, micro, and millifluidics can still serve as valuable tools for many investigations. GLUBscope is of interest in this context for the biomechanics and hydrodynamics of aquatic organisms. Broader access to resources (in particular imaging) and closer investigation of the aquatic organisms could provide an avenue for exciting discoveries.

## CRediT authorship contribution statement

**Brian T. Le:** Writing – original draft, Visualization, Validation, Investigation. **Katherine M. Auer:** Writing – original draft, Visualization, Validation, Investigation. **David A. Lopez:** Writing – original draft, Visualization, Validation, Investigation. **Justin P. Shum:** Writing – original draft, Visualization, Validation, Investigation. **Brian Suarsana:** Writing – original draft, Visualization, Validation, Investigation. **Ga-Young Kelly Suh:** Writing – original draft, Visualization, Validation, Supervision, Resources, Project administration, Methodology, Investigation. **Per Niklas Hedde:** Writing – original draft, Visualization, Validation, Resources, Methodology, Investigation, Funding acquisition, Conceptualization. **Siavash Ahrar:** Writing – review & editing, Writing – original draft, Visualization, Validation, Supervision, Resources, Project administration, Investigation, Funding acquisition, Formal analysis, Data curation, Conceptualization.

## Declaration of competing interest

The authors declare that they have no known competing financial interests or personal relationships that could have appeared to influence the work reported in this paper.

## Appendix A Supplementary data

MMC S1 Supplementary materials include videos, bill of materials, and design files.
