# Supplementary material for: Orthogonal-view microscope for the biomechanics investigations of aquatic organisms
Source: HardwareX. 2024 Apr 22;18:e00533. doi: 10.1016/j.ohx.2024.e00533 (PMC11070628; doi:10.1016/j.ohx.2024.e00533)
Supplement: MMC S1 — Supplementary materials include videos, bill of materials, and design files. [file mmc1.zip › supplementary.pdf]

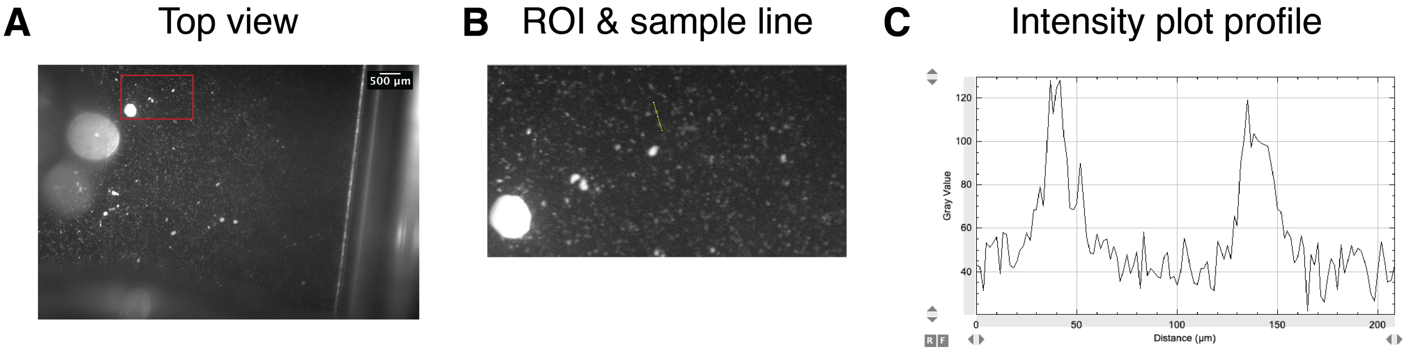

Figure 6: **Supplementary Figure-1: Fluorescent imaging.** (A) Green fluorescent beads (5 microns) were mixed in water and vortexed inside a cuvette (3.5 mL volume and path length 1 cm). An off-the-shelf Green LED was used to illuminate the sample. (B) A region of interest (ROI) and a sample line for intensity measurements were selected. (C) The intensity profile along the sample line indicates the bead clusters. The signal-to-noise could be improved via a brighter excitation light source and by reducing background noise.

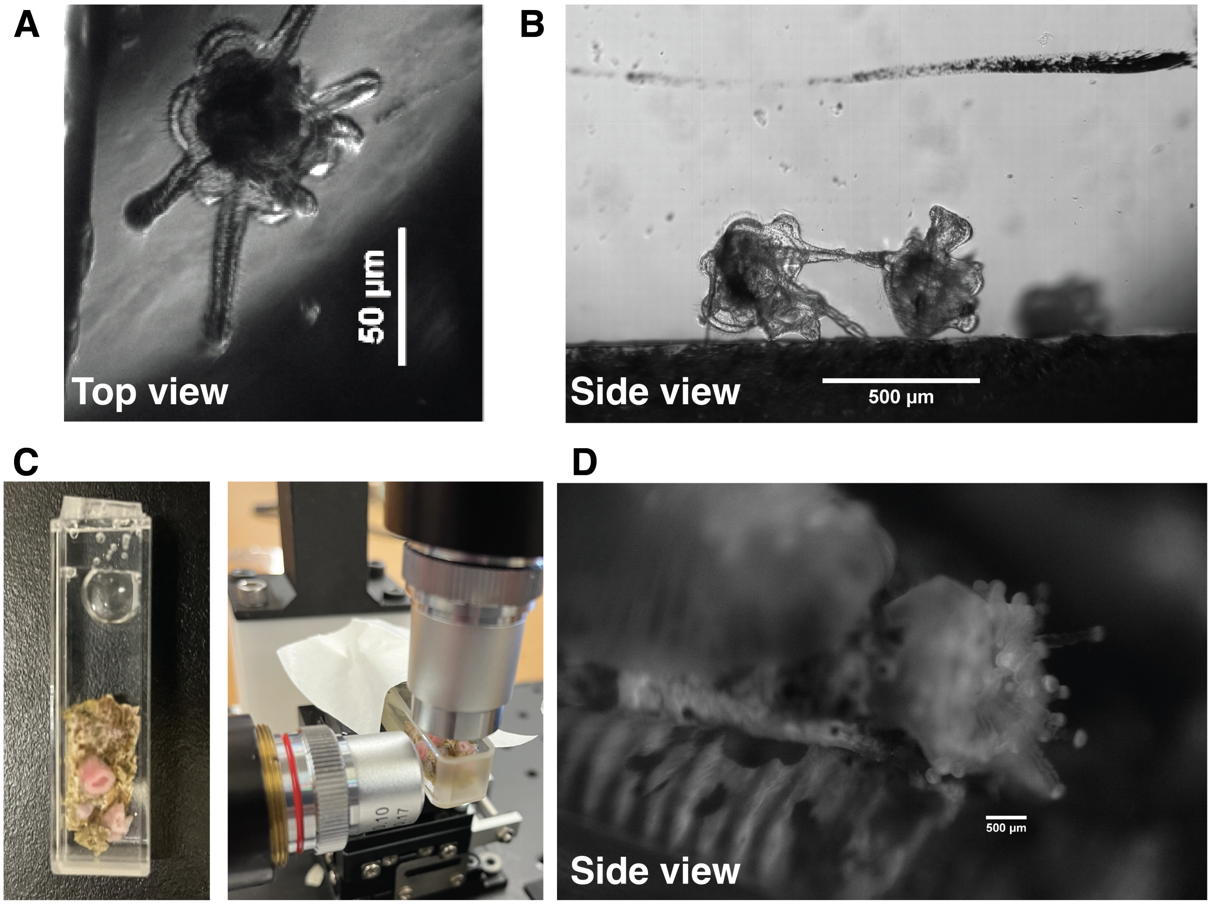

Figure 7: **Supplementary Figure-2: GLUBscope - case studies**. (A-B) top and side views of sand dollar larvae (C) Sea anemone attached to a rock inside the sample holder and on the stage. (D) The sea anemone was imaged from the side view using GLUBscope.
