# Supplementary material for: Orthogonal-view microscope for the biomechanics investigations of aquatic organisms
Source: HardwareX. 2024 Apr 22;18:e00533. doi: 10.1016/j.ohx.2024.e00533 (PMC11070628; doi:10.1016/j.ohx.2024.e00533)
Supplement: MMC S1 — Supplementary materials include videos, bill of materials, and design files. [file mmc1.zip › GLUB_bill of materials .pdf]

Following is the bill of materials for the project.

Mechanical hardware

1. SM1A3 - ThorLabs

   How many: 1 or 2
   Cost per: $19.43 % Adapter with External SM1 Threads and Internal RMS Threads

2. SM1L05 - ThorLabs

   How many:1 or 2
   Cost per: $13.62 % Stackable lens mount for 1" depth 1/2

3. SM1L03 - ThorLabs

   How many:1 or 2 Cost per: $13.15 % 1" Stackable Lens Tube

4. SM1L10 - ThorLabs

   How many: 1 or 2 Cost per: $15.41 % Stackable Lens Mount For 1" Optic-Usable Depth 1"

5. SM1L30 - ThorLabs

   How many: 1 or 2 Cost per: $29.23 % SM1 Lens Tube, 3.00" Thread Depth, One Retaining Ring Included

6. XYZ Axis Micromanipulator

   How many: 1 Cost per: $125 % XYZ Axis Manual Precision Linear Stage 40x40mm Trimming Bearing Tuning Platform Sliding Table

7. [MB8- Thor labs][] - ThorLabs

   How many: 1 Cost per: % Aluminum Breadboard 8" x 8" x 1/2", 1/4"-20 Taps. There are many alternatives for this part.

8. [¼”-20 Screws]

Electronic components

1. LED - 3W Aluminum PCB (Cool White)

   How many: 1 or 2 Cost per: $8.95 per pack of 5 % LED for brightfield illuminations

2. FLIR camera - Blackfly S USB3

   How many: 0, 1 or 2 Cost per: ~$700 Note: we used two in certain experiments. Models: BFS-U3-200S6C-C: 20 MP, 18 FPS, Sony IMX183, Color and Mono. Note: Does not include

the cable. We used USB 3.1 Locking
Cable, 3m

3. Arducam, 12MP IMX477 6mm CS-Mount Lens

   How many: 0, 1 or 2 Cost per: ~$120 Note: we used one in certain experiments. Note: includes the cable.

4. [Electrical Breadboard]

   How many: 0 or 1 Note: to wire the LEDs and power them. Cost: ~ $5

5. [9V battery or power supply]

6. [Jumper wires and resistors]

   Note: 350 ohm resistors were used

Optical components

1. Lens f = 50 mm, Ø1" Achromatic Doublet, ARC: 400 - 700 nm

   How many: 2 Cost per: $87.50 Note: One for each light path

2. [Objectives: Link-1 Link-2]

   How many: 2 Cost: $ 70-150 Better objectives can be used in the system. Various objectives were used in this study. Typically a 4X for the top view and a 10X for the side view (10×, NA 0.25)

**Sample holder **

1. [4 Clear Windows Fluorometer Cell Cuvette]

   How many: 1 (Pack of 2) Note: LAB4US Fluorescence Glass Cuvette (2pcs) Fluorescence Cuvettes for Spectrophotometer Cuvettes. 10 mm pathlength 1cm, 3.5ml, 4 Clear Windows

---

Other - optional parts

1. [Irwin tools quick-grip c-clamp]

   How many: 1 or 2 1 ½-inch, 1 ½ - inch (2025101). If needed for added security for Arducam cameras were needed.

2. [Emission filter (500 nm long pass, ET500lp)]

   Vendor: Chroma - ET500lp How many: 0, 1 or 2 % Note: Not needed for brightfield imaging. In our study it was included for future fluorescence imaging applications

3. [LED - Green LED]

   How many: 1 or 2 LED for optional fluorescence imaging.

Note-1: Costs are all at the time of purchase and should be considered a baseline estimation.

Note-2: List of 3D printed parts are provided via a different folder.
